# Risk Factor Predictors for Developing Epilepsy in Cerebral Palsy Patients in a Tertiary Hospital in Saudi Arabia: A Retrospective Study

**DOI:** 10.7759/cureus.59980

**Published:** 2024-05-09

**Authors:** Reem Alyoubi, Ammar Mirza, Fadi Busaleh, Odai W Ashgar, Abdulrahman A Alamoudi, Ahmad M Alnoiqy, Faisal A Alghamdi, Muhnnad A AlGhamdi, Ahlam Mazi, Huda Alyahyawi

**Affiliations:** 1 Neurology, King Abdulaziz University Hospital, Jeddah, SAU; 2 Faculty of Medicine, King Abdulaziz University, Jeddah, SAU; 3 Pediatrics, Maternity and Children Hospital (MCH), Al-Ahsa, SAU; 4 Pediatric Neurology, Faculty of Medicine, King Abdulaziz University, Jeddah, SAU; 5 College of Medicine, Faculty of Medicine, King Abdulaziz University, Jeddah, SAU; 6 College of Medicine, King Saud bin Abdulaziz University for Health Sciences, Jeddah, SAU; 7 Pediatrics, King Abdulaziz University Hospital, Jeddah, SAU; 8 Pediatrics, King Abdulaziz University, Jeddah, SAU; 9 Psychiatry, King Abdulaziz University Hospital, Jeddah, SAU

**Keywords:** cerebral palsy types, cerebral palsy, childhood epilepsy, epilepsy education, pediatric seizure, pediatric

## Abstract

Background

Cerebral palsy (CP) is a major cause of childhood motor impairment worldwide. The prevalence of CP related to preterm births has increased consistently. Perinatal hypoxic-ischemic encephalopathy, intra- or periventricular haemorrhage, cerebral dysgenesis and intracranial infections are among the factors contributing to CP onset.

Several studies have explored epilepsy-related morbidity among children with CP, finding notable correlations between the two conditions. Worldwide, there are multiple studies highlighting the high prevalence of epilepsy among children with CP and its association with specific CP subtypes and neurologic insults. However, research on the risk factors for epilepsy in CP children is limited, particularly in the Middle East and Saudi Arabia.

Aim

This study aims to address this gap by analysing potential prenatal, antenatal, and postnatal risk factors associated with epilepsy development in children with CP.

Methods

A retrospective cohort analysis of 152 children aged 1-14 years diagnosed with CP at King Abdulaziz University Hospital, Jeddah, Saudi Arabia, was conducted.

Results

The study showed a significant prevalence of epilepsy (68.4%), with generalised seizures being the most common type. Quadriplegia was notably common among CP children with epilepsy, indicating a potential correlation between motor impairment severity and epilepsy risk. Furthermore, CP children with epilepsy exhibited a higher prevalence of co-morbidities, emphasising the multifaceted nature of this condition. Perinatal and neonatal factors, such as hypoxic events, mechanical ventilation, perinatal asphyxia, neonatal convulsions, and microcephaly, were identified as significant risk factors for epilepsy in children with CP. While speech and hearing disorders were present in CP children with and without epilepsy, a slightly higher prevalence of impaired speech was observed in those with epilepsy. However, the difference between the two groups was not significant.

Conclusion

This study provides valuable insights into the epidemiology, clinical characteristics and potential risk factors associated with epilepsy among children diagnosed with CP in Saudi Arabia. The findings underscore the complexity of managing epilepsy in this population and highlight the need for further research to elucidate the underlying mechanisms and support the development of targeted interventions to improve patient outcomes.

## Introduction

Cerebral palsy (CP) manifests as both neurodevelopmental and physical disabilities that emerge during childhood. It is a significant cause of motor impairment among children, with an incidence ranging from two to three cases per 1000 live births worldwide, 1.8 cases per 1000 live births in Arabic-speaking countries and 1.6 cases per 1000 live births in Saudi Arabia. Notably, the prevalence of CP associated with preterm births has increased consistently. Various factors contribute to the onset of CP, including perinatal hypoxic-ischemic encephalopathy, intra- or periventricular haemorrhage, cerebral dysgenesis and intracranial infection [1-3]. 

Several studies have investigated epilepsy-related morbidity among CP patients, aiming to identify associated risk factors, as CP is significantly correlated with epilepsy. For example, Sadowska et al. conducted a study on 181 children with CP, reporting that 56.35% were diagnosed with epilepsy, with a higher prevalence observed among children with tetraplegic CP [4]. Additionally, Carlsson et al. found that children with CP stemming from central nervous system malformations, infections or grey matter damage were at higher risk of developing seizures [5].

Despite these findings, research on the risk factors for epilepsy in children with CP is limited in the Middle East specifically in Saudi Arabia. This study aims to fill this research gap by analysing potential prenatal, antenatal and postnatal risk factors associated with the development of epilepsy in children with CP.

## Materials and methods

Study design

A descriptive retrospective record review was conducted using King Abdulaziz University Hospital's electronic file system. Data were collected by reviewing reports of paediatric patients diagnosed with CP between 2009 and 2022. Patients’ files were accessed through the databases of the paediatric neurology and neuroradiology department at King Abdulaziz University Hospital, a tertiary care centre in Jeddah, Saudi Arabia. Ethical approval for the study was obtained from the hospital’s research and ethics committee (reference number 711-23).

Study population

We conducted a retrospective analysis of 152 children aged 1-14 years who were diagnosed with CP at King Abdulaziz University Hospital in Jeddah, Saudi Arabia.

Inclusion criteria

The inclusion criteria were as follows: age 1-14 years at the time of enrolment, a diagnosis of CP confirmed by an experienced paediatric neurologist and available neuroimaging results, including magnetic resonance imaging (MRI) or computed tomography (CT) scans.

Exclusion criteria

The exclusion criteria were as follows: age younger than one year or older than 14 years, a lack of neuroimaging results, clinical features indicative of progressive encephalopathies or the presence of metabolic inborn errors.

CP cases were classified into six different types: (i) spastic quadriplegia: all four limbs affected, (ii) spastic hemiplegia: affecting only a single hemi-body part with increased tone in flexor muscles, (iii) spastic diplegia: mainly affecting both lower limbs, with possible upper limb involvement, but with tone abnormalities less severe than those of the lower limbs, (iv) dyskinetic: involving dystonia (faulty posture with enhanced muscle tone) and choreoathetosis (quick, uncontrolled, twisting movements with hypotonia),(v) ataxic: characterised by hypotonia and loss of coordination and (vi) mixed type [6]. 

MRI brain images were classified according to the Magnetic Resonance Imaging Classification System (MRICS) as maldevelopment, predominant white matter injury, predominant grey matter injury, miscellaneous and normal. Movement disorder severity was classified according to the Gross Motor Function Classification Scale (GMFCS), ranging from most able (level I) to most disabled (level V) [7].

Data analysis

The data were collected, reviewed and then fed into IBM SPSS Statistics for Windows, Version 26 (Released 2019; IBM Corp., Armonk, New York, United States). All statistical analyses were two-tailed with an alpha level of 0.05, and p-values less than or equal to 0.05 were considered significant. Descriptive analysis was conducted by providing frequency distributions and percentages for categorical variables, while quantitative variables were represented as means and standard deviations. Cross-tabulation was employed to illustrate different factors associated with epilepsy among CP cases (child data and perinatal factors) using Pearson’s Chi-square test and the exact probability test for small frequency distributions.

## Results

A total of 152 children with CP were included in this study. Of these children, 104 (68.4%) were diagnosed with epilepsy, while 48 (31.6%) were epilepsy free. The age of the children with CP was 1-14 years, with a mean age of 7.1 ± 4.2 years, and 86 (57%) were male. The most prevalent type of CP was quadriplegia (57.9%), followed by diplegia (12.5%), unspecified spasticity (7.9%) and hemiplegia (6.6%). MRI findings revealed normal results in 82 cases (53.9%), while miscellaneous findings were observed in 46 (30.3%), white matter lesions (10.5%), maldevelopment (7.9%) and grey matter lesions (7.2%). Of the participants, 113 (74.3%) had co-morbidities, with 70 (46.1%) experiencing global developmental delay and 10 (6.6%) having a family history of epilepsy.

**Figure 1:**
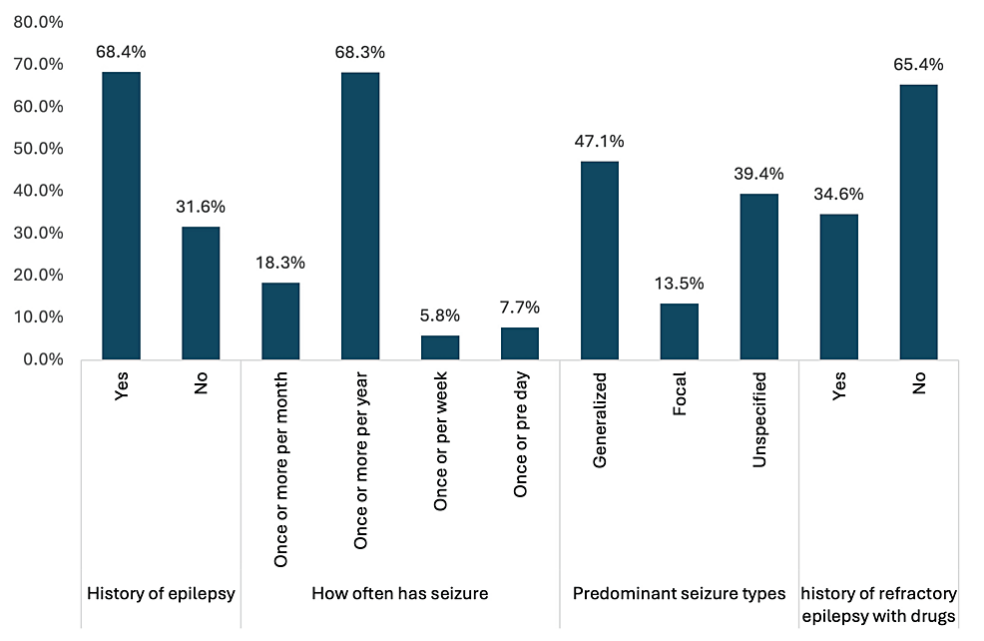
Prevalence, Frequency and Types of Epilepsy Among Study Cases with CP CP: Cerebral palsy

Regarding the prevalence, frequency, and types of epilepsy among the study cases with CP, 104 (68.4%) were diagnosed with epilepsy, which manifested as generalised (47.1%), focal (13.5%) and unspecified (39.4%) types. Furthermore, it has been observed in CP with epilepsy that about 68.3% of the cases had one or more seizure attacks per year, while 18.3% experienced attacks once per month, and 7.7% experienced them once per day. Additionally, 34.6% exhibited refractory epilepsy despite treatment with medication. Figure 1 shows the prevalence and frequency of each study group case. 

Table 1 shows the characteristics of the study group. A total of 67 (64.4%) children in the subgroup with CP and epilepsy had quadriplegia compared to 21 (43.8%) in the epilepsy-free subgroup, and 10 (20.8%) children in the epilepsy-free subgroup had diplegia compared to nine (8.7%) in the subgroup with CP and epilepsy (p=.024). Additionally, 85 (81.7%) children in the subgroup with CP and epilepsy had other co-morbidities compared to 28 (58.3%) in the epilepsy-free subgroup (p=.002). 

**Table 1:** Characteristics of the Study Group (Children with CP and Epilepsy) P: Pearson X2 test; ^: Exact probability test; * p < 0.05 (significant) CP: Cerebral palsy

		Total Group with CP (n=152)		Subgroup with CP and Epilepsy (n=104)		Epilepsy-Free Subgroup (n=48)		p-value
		No	%	No	%	No	%	
Age at enrolment in the study	< 5	50	32.9%	31	29.8%	19	39.6%	.460
	5–9	52	34.2%	36	34.6%	16	33.3%	
	10–14	50	32.9%	37	35.6%	13	27.1%	
Gender	Male	86	57.0%	59	56.7%	27	57.4%	.934
	Female	65	43.0%	45	43.3%	20	42.6%	
Type of cerebral palsy	Quadriplegia	88	57.9%	67	64.4%	21	43.8%	.024*
	Hemiplegia	10	6.6%	7	6.7%	3	6.3%	
	Diplegia	19	12.5%	9	8.7%	10	20.8%	
	Unspecified spasticity	12	7.9%	9	8.7%	3	6.3%	
	Dyskinetic	1	.7%	1	1.0%	0	0.0%	
	Mixed	3	2.0%	3	2.9%	0	0.0%	
	NA	19	12.5%	8	7.7%	11	22.9%	
Gross Motor Function Classification Scale (GMFCS)	I	4	22.2%	2	22.2%	2	22.2%	.539^
	II	2	11.1%	0	0.0%	2	22.2%	
	III	1	5.6%	1	11.1%	0	0.0%	
	IV	2	11.1%	1	11.1%	1	11.1%	
	V	9	50.0%	5	55.6%	4	44.4%	
MRI findings	Normal	82	53.9%	51	49.0%	31	64.6%	.167^
	Maldevelopment	12	7.9%	10	9.6%	2	4.2%	
	White matter lesion	16	10.5%	13	12.5%	3	6.3%	
	Gray matter lesion	11	7.2%	7	6.7%	4	8.3%	
	Miscellaneous	46	30.3%	35	33.7%	11	22.9%	
Co-morbidities	Yes	113	74.3%	85	81.7%	28	58.3%	.002*
	No	39	25.7%	19	18.3%	20	41.7%	
Hypothyroidism	Yes	16	10.5%	14	13.5%	2	4.2%	.083^
	No	136	89.5%	90	86.5%	46	95.8%	
Hydrocephalus	Yes	11	7.2%	8	7.7%	3	6.3%	.750
	No	141	92.8%	96	92.3%	45	93.8%	
Failure to thrive	Yes	12	7.9%	10	9.6%	2	4.2%	.247^
	No	140	92.1%	94	90.4%	46	95.8%	
Global developmental delay	Yes	70	46.1%	51	49.0%	19	39.6%	.277
	No	82	53.9%	53	51.0%	29	60.4%	
Family history of epilepsy	Yes	10	6.6%	9	8.7%	1	2.1%	.129^
	No	142	93.4%	95	91.3%	47	97.9%	

Table 2 shows perinatal, neonatal and infant-related risk factors for epilepsy in CP. A total of 32 (30.8%) children in the subgroup with CP and epilepsy required mechanical ventilators compared to six (12.5%) in the epilepsy-free subgroup (p=.016). Likewise, 18 (17.3%) children in the subgroup with CP and epilepsy experienced neonatal convulsions compared to only one in the epilepsy-free subgroup (P=.001). Microcephaly was observed in five (4.8%) of the children in the subgroup with CP and epilepsy compared to seven (14.6%) in the epilepsy-free subgroup (p=.038), and 44 (42.3%) children in the subgroup with CP and epilepsy had a history of asphyxia compared to 12 (25%) in the epilepsy-free subgroup (p=.040). 

**Table 2:** Perinatal, Neonatal and Infant-Related Risk Factors for Epilepsy in CP P: Pearson X2 test; ^: Exact probability test; * p < 0.05 (significant) CP: Cerebral palsy

		Total Group with CP (n=152)		Subgroup with CP and Epilepsy (n=104)		Epilepsy-Free Subgroup (n=48)		p-value
		No	%	No	%	No	%	
Birth week	< 28 weeks	8	5.3%	5	4.8%	3	6.3%	.326^
	28–32 weeks	6	3.9%	2	1.9%	4	8.3%	
	33–38 weeks	12	7.9%	7	6.7%	5	10.4%	
	> 38 weeks	21	13.8%	15	14.4%	6	12.5%	
Type of delivery	NVD	29	19.1%	20	19.2%	9	18.8%	.903
	Caesarean section	25	16.4%	18	17.3%	7	14.6%	
	NA	98	64.5%	66	63.5%	32	66.7%	
Apgar score at the first minute	0–3	3	25.0%	3	33.3%	0	0.0%	.135^
	4–7	6	50.0%	5	55.6%	1	33.3%	
	8–10	3	25.0%	1	11.1%	2	66.7%	
Apgar score at the fifth minute	0–3	1	8.3%	1	11.1%	0	0.0%	.368^
	4–7	8	66.7%	5	55.6%	3	100.0%	
	8–10	3	25.0%	3	33.3%	0	0.0%	
NICU stay	<10 days	13	8.6%	11	10.6%	2	4.2%	.279^
	10–30 days	31	20.4%	24	23.1%	7	14.6%	
	>30 days	18	11.8%	12	11.5%	6	12.5%	
	NA	90	59.2%	57	54.8%	33	68.8%	
Attachment to mechanical ventilator	Yes	38	25.0%	32	30.8%	6	12.5%	.016*
	No	114	75.0%	72	69.2%	42	87.5%	
Neonatal convulsion	Yes	19	12.5%	18	17.3%	1	2.1%	.001*
	No	47	30.9%	24	23.1%	23	47.9%	
	NA	86	56.6%	62	59.6%	24	50.0%	
Microcephaly	Yes	12	7.9%	5	4.8%	7	14.6%	.038*
	No	140	92.1%	99	95.2%	41	85.4%	
History of asphyxia	Yes	56	36.8%	44	42.3%	12	25.0%	.040*
	No	96	63.2%	60	57.7%	36	75.0%	
History of dysphagia	Yes	12	8.4%	8	8.2%	4	8.7%	.120
	No	46	32.2%	26	26.8%	20	43.5%	

**Figure 2:**
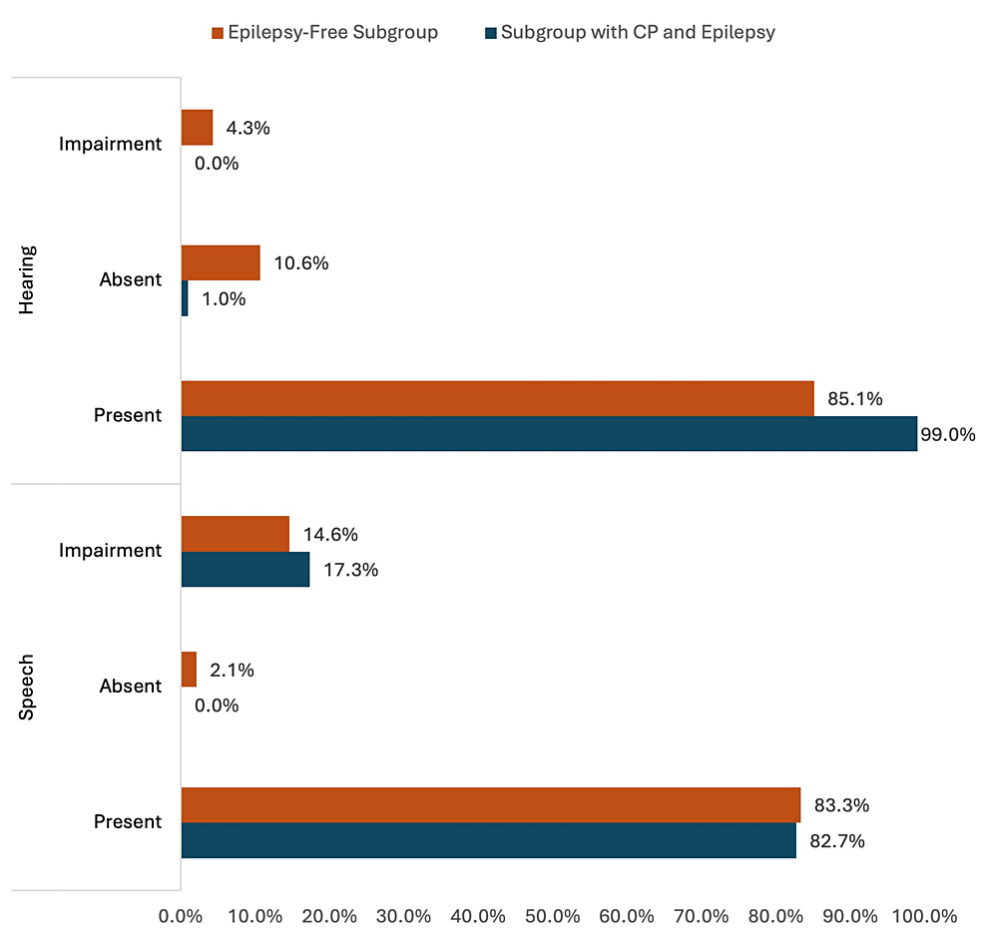
Speech and Hearing Disorders Among Study Groups CP: Cerebral palsy

Figure 2 depicts speech and hearing disorders among the study groups. In the subgroup with CP and epilepsy, 17.3% of the children exhibited impaired speech compared to 14.6% in the epilepsy-free subgroup. None of the children in the subgroup with CP and epilepsy had impaired hearing, compared to 4.3% in the epilepsy-free subgroup.

## Discussion

CP refers to a static brain injury that leads to permanent motor dysfunction. Although the condition itself is not neurodegenerative, the clinical manifestations may change over time as the central nervous system matures. It can lead to disabilities that go beyond motor impairments. Epilepsy is one of the main complications driving the investigation of potential risk factors for both CP and epilepsy [8,9].

Prevalence and characteristics of epilepsy 

Our findings indicate a substantial prevalence of epilepsy among children with CP, with 68.4% of the study cohort diagnosed with epilepsy. This finding aligns with prior research highlighting the notable comorbidity between CP and epilepsy. Generalised seizures were the predominant seizure type, followed by unspecified and focal seizures. Importantly, a significant proportion of CP children with epilepsy reported frequent seizures, with 68.3% experiencing one or more attacks per year. Additionally, a notable subset demonstrated refractory epilepsy despite medication, underscoring the challenges in seizure management within this population. These findings are consistent with both local and global studies showing that epilepsy is highly prevalent among CP patients [2,4,10,11].

Correlation with CP subtypes and co-morbidities

Our study revealed an intriguing association between specific CP subtypes and the presence of epilepsy. Quadriplegia was notably common among CP children with epilepsy, suggesting a potential correlation between the severity of motor impairment and the risk of epilepsy development. Furthermore, a higher prevalence of co-morbidities was observed among CP children with epilepsy, highlighting the intricate clinical presentation and multifaceted nature of this condition. This result corresponds to findings from previous local studies [3,8,9].

Perinatal and neonatal risk factors

Our study underscores the pivotal role of perinatal and neonatal factors in the development of epilepsy among children with CP. In particular, hypoxic events emerged as significant risk factors predisposing CP patients to epilepsy. Specifically, mechanical ventilation during the neonatal period was identified as a notable risk factor, with a higher prevalence among CP children with epilepsy. Additionally, a history of perinatal asphyxia was more commonly observed among CP children with epilepsy, highlighting the potential contribution of hypoxic-ischemic injury to epilepsy pathogenesis in this population. Similarly, a history of neonatal convulsions and microcephaly was more prevalent among CP children with epilepsy, emphasising the importance of early detection and intervention for these high-risk individuals. These findings are consistent with previous research studies globally but not confined locally [2-4,12,13].

Speech and hearing disorders

Although both speech and hearing impairments were found in children with CP, regardless of whether they had epilepsy or not, our study shows a slightly higher incidence of speech impairment in CP children with epilepsy. However, this difference was not statistically significant. Our results align with those of Reid et al. regarding the association between epilepsy and hearing loss but in contrast with Weir et al., who reported a significantly higher prevalence of hearing loss at 39% [14,15]. Despite the lack of statistical significance in our study, it emphasises the importance of thorough assessment and management of communication disorders in CP children, especially those with epilepsy, to improve overall developmental outcomes.

## Conclusions

In conclusion, our study provides valuable insights into the epidemiology, clinical characteristics and potential risk factors associated with epilepsy among children diagnosed with CP in Saudi Arabia and the Middle East. The high prevalence of epilepsy, its correlation with specific CP subtypes and co-morbidities and the identified perinatal and neonatal risk factors highlight the complexity of managing epilepsy in this population. Further research is needed to elucidate the underlying mechanisms and to develop targeted interventions aimed at improving the quality of life and long-term outcomes of CP children affected by epilepsy because most studies are retrospective and there is a lack of prospective studies.
